# Differential genome evolution and speciation of *Coix lacryma-jobi* L. and *Coix aquatica* Roxb. hybrid guangxi revealed by repetitive sequence analysis and fine karyotyping

**DOI:** 10.1186/1471-2164-15-1025

**Published:** 2014-11-25

**Authors:** Zexi Cai, Huijun Liu, Qunyan He, Mingwei Pu, Jian Chen, Jinsheng Lai, Xuexian Li, Weiwei Jin

**Affiliations:** National Maize Improvement Center of China, Beijing Key Laboratory of Crop Genetic Improvement, Coordinated Research Center for Crop Biology, China Agricultural University, Beijing, 100193 China; Zhejiang Academy of Agricultural Science, Hangzhou, 310021 China; State Key Laboratory of Agro-biotechnology and National Maize Improvement Center, Department of Plant Genetics and Breeding, China Agricultural University, Beijing, 100193 China; Department of Plant Nutrition, China Agricultural University, Beijing, 100193 China

**Keywords:** *Coix*, Next-generation sequencing, Repeat element, Genome structure, Karyotyping, Polyploidy, Evolution

## Abstract

**Abstract:**

**Background:**

*Coix*, *Sorghum* and *Zea* are closely related plant genera in the subtribe *Maydeae. Coix* comprises 9–11 species with different ploidy levels (2n = 10, 20, 30, and 40). The exclusively cultivated *C. lacryma-jobi* L. (2n = 20) is widely used in East and Southeast Asia for food and medicinal applications. Three fertile cytotypes (2n = 10, 20, and 40) have been reported for *C. aquatica* Roxb. One sterile cytotype (2n = 30) closely related to *C. aquatica* has been recently found in Guangxi of China. This putative hybrid has been named *C. aquatica* HG (Hybrid Guangxi). The genome composition and the evolutionary history of *C. lacryma-jobi* and *C. aquatica* HG are largely unclear.

**Results:**

About 76% of the genome of *C. lacryma-jobi* and 73% of the genome of *C. aquatica* HG are repetitive DNA sequences as shown by low coverage genome sequencing followed by similarity-based cluster analysis. In addition, long terminal repeat (LTR) retrotransposable elements are dominant repetitive sequences in these two genomes, and the proportions of many repetitive sequences in whole genome varied greatly between the two species, indicating evolutionary divergence of them. We also found that a novel 102 bp variant of centromeric satellite repeat CentX and two other satellites only appeared in *C. aquatica* HG. The results from FISH analysis with repeat probe cocktails and the data from chromosomes pairing in meiosis metaphase showed that *C. lacryma-jobi* is likely a diploidized paleotetraploid species and *C. aquatica* HG is possibly a recently formed hybrid. Furthermore, *C. lacryma-jobi* and *C. aquatica* HG shared more co-existing repeat families and higher sequence similarity with *Sorghum* than with *Zea*.

**Conclusions:**

The composition and abundance of repetitive sequences are divergent between the genomes of *C. lacryma-jobi* and *C. aquatica* HG*.* The results from fine karyotyping analysis and chromosome pairing suggested diploidization of *C. lacryma-jobi* during evolution and *C. aquatica* HG is a recently formed hybrid. The genome-wide comparison of repetitive sequences indicated that the repeats in *Coix* were more similar to those in *Sorghum* than to those in *Zea*, which is consistent with the phylogenetic relationship reported by previous work.

**Electronic supplementary material:**

The online version of this article (doi:10.1186/1471-2164-15-1025) contains supplementary material, which is available to authorized users.

## Background

*Coix lacryma-jobi* L. (2n = 20), also commonly called adlay (Job’s tears), is widely cultivated as a food and medicine plant in East and Southeast Asian countries[1]. Its grain has the highest protein content among cereal crops[2], and its seed extracts are used to treat several diseases[3, 4]. Previous research on *Coix* has focused mainly on its medicinal components and their efficacy[5, 6]. Genetic and genomic studies on *Coix* are sparse, although there are some reports on cytogenetic analysis[7–9], genetic linkage map development[10], construction of BAC libraries[11], and isolation of prolamin gene families[12, 13].

The genus *Coix* belongs to *Maydeae* of Poaceae family and is the closest group to the genera *Zea*, *Tripsacum,* and *Sorghum*[14]. The places of the origin of the three genera are very different. *Zea* originates from America, and *Sorghum* originates from Africa, while the genus *Coix* is indigenous to Southeast Asia[1]. Previous studies have shown that *Coix* comprises of 9–11 species with different levels of ploidy. Tetraploids (2n = 20) predominate, including the exclusively cultivated *Coix lacryma-jobi* L., while diploids (2n =10), hexaploids (2n = 30), and octoploids (2n = 40) with a basic chromosome number of x = 5 are less frequent[15]. *Coix aquatica* Roxb. has been reported to have three fertile cytotypes (2n = 10, 20 and 40). A sterile cytotype (2n = 30) of very close botanical relationship to *C. aquatica* was found in southwest China[16]. The results from genomic *in situ* hybridization (GISH) demonstrated that 20 out of its 30 chromosomes are highly homologous to the chromosomes of *C. lacryma-jobi*[7]. The genomic and evolutionary information of *C. lacryma-jobi* and this sterile *C. aquatica* remains elusive. Furthermore, the phylogenetic relationship between *Coix* and *Zea*/*Sorghum* is still unclear. Comparative analysis of genomic DNA sequences may contribute to answer those questions.

The majority of the genomic sequences in higher eukaryotes are repetitive elements including transposable elements and satellite repeats[17]. Large-scale accumulation of satellites and transposable elements serves as a major driving force for genome expansion[18, 19]. Most long terminal repeat (LTR) retrotransposable elements disperse throughout plant chromosomes, while some elements locate in specific regions of chromosomes[20]. Satellite DNA sequences, consisting of short tandem repeats, share a common structural feature[21]. They are usually located in specific chromosomal regions and may be broadly classified as centromeric, subtelomeric, or intercalary repetitive sequences[22]. Satellite repeats are very powerful tools for chromosome identification, chromosome karyotyping, and comparative genome analysis[23, 24]. Because of similar chromosome lengths and/or arm ratios and lack of sufficient landmarks, adequate identification of *Coix* chromosome pairs remains challenging. Therefore, identification and characterization of major repetitive sequences are essential for understanding the organization and structure of *Coix* genomes.

In this study, we sequenced ~6 Gb genomic sequences of *C. lacryma-jobi* and 12 Gb genomic sequences of *C. aquatica* HG, and found highly dynamic nature of these two genomes during evolution. Our karyotyping analysis revealed that *C. lacryma-jobi* is a diploidized paleotetraploid species, and *C. aquatica* HG is recently formed hybrid between *C. lacryma-jobi* and a distantly related species. Comparative repeat sequence analysis supported that *Coix* is closer to *Sorghum* than to *Zea*. The findings in this study advance our understanding of *Coix* genome evolution and shed new light on the genomics of the Poaceae family.

## Results

### Low-coverage sequencing of *C. lacryma-jobi*and *C. aquatica*HG genomes and repetitive sequence analysis

To better understand the *Coix* genus at the genome level, we determined the genome size of the two *Coix* species using flow cytometry with the maize inbred line B73 (approximately 2,300 Mb) as a reference genome (Additional file1: Figure S1). The genome size of *C. lacryma-jobi* was approximately 1,684 Mb (Table 1), similar to the previous report[25]. *C. aquatica* HG had a genome of approximately 2,335 Mb (Table 1), about 1.4-fold of the *C. lacryma-jobi* genome.

**Table 1:** **Size estimation and sequencing of** ***C. lacryma-jobi*** **and** ***C. aquatica*** **HG genomes**

Species	Chromosome number (2n)	Genome size (Gb)	Number of reads	Total read length (Mb)	Genome coverage
*C. lacryma-jobi*	20	1.684	61,869,688	6186.97	1.84×
*C. aquatica*	30	2.335	128,853,294	12885.33	2.76×

We sequenced (Table 1) and used cluster-based repeat identification and classification method[26] to analyze the data. Except Penelope retrotransposon and P transposon superfamilies[27], all other plant transposable elements and repetitive DNA were detected in these two species, including LTR and LINE/SINE retrotransposons, Mutator and En-Spm transposons, satellites, and rDNA. The proportion of total repetitive sequences in whole genome is 75.54% in *C. lacryma-jobi* and 72.88% in *C. aquatica* HG. The proportion of different types of repetitive DNA was displayed in Table 2.

**Table 2:** **Repetitive sequences and their proportions in** ***C. lacryma-jobi*** **and** ***C. aquatica*** **HG**

Proportion in two genomes (%)		
Repeat family	*C. lacryma-jobi*	*C. aquatica*
Retrotransposon	69.08	63.83
Ty1/Copia	39.57	31.12
Ty3/Gypsy	29.11	32.56
Unclassified LTR	0.28	0.12
LINE/SINE	0.12	0.03
Transposon	4.38	3.30
hAT	0.03	0.06
Mutator	0.57	0.37
RC/Helitron	0.02	0.04
En-Spm	3.64	2.42
PIF-Harbinger	0.09	0.24
Tc1-Mariner	0.01	0.14
Other	0.02	0.03
rDNA	0.43	0.56
snRNA	ND	ND
Satellite	0.60	4.89
Unclassified	1.05	0.30
Total	75.54	72.88

Note: snRNA, small noncoding RNA; ND, not detected.

We found that retroelements occupied 69.08% of the *C. lacryma-jobi* genome and 63.83% of the *C. aquatica* HG genome (Table 2). The two dominant families of retroelements are Ty1/Copia and Ty3/Gypsy retrotransposons. The Ty1/Copia occupied 39.57% of the *C. lacryma-jobi* genome and 31.12% of the *C. aquatica* HG genome, while the Ty3/Gypsy occupied 29.11% and 32.56%, respectively. The most abundant type I transposon was the Spm class of transposons, and the Mutator was also enriched in *Coix*.

**Figure 1:**
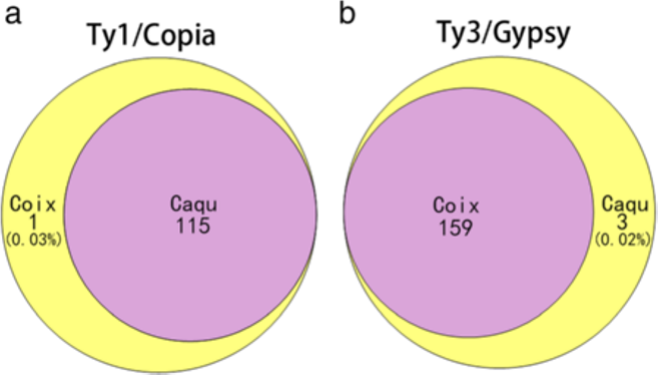
**Ty1/Copia and Ty3/Gypsy cluster distribution in** ***C. lacryma-jobi*** **and** ***C. aquatica*** **HG.** Ty1/Copia and Ty3/Gypsy cluster distribution in *C. lacryma-jobi* and *C. aquatica* HG. **(a)** *C. lacryma-jobi* and *C. aquatica* HG had 115 Ty1/Copia clusters in common, and only one distinct cluster was detected only in *C. lacryma-jobi*. **(b)** *C. lacryma-jobi* and *C. aquatica* HG shared 159 common Ty3/Gypsy clusters, and 3 clusters were detected only in *C. aquatica* HG. The percentage represents the proportion of corresponding clusters in the genome.

We believed that the abundance of many repeats varied greatly between these two species. To test this hypothesis, we conducted a comparative analysis by combining reads of these two species and performing cluster-based repeat identification and classification on the combined reads[28, 29]. In spite of lacking statistical examination on these data, we found that CL1, a Ty1/Copia member, occupied 3.44% of the *C. lacryma-jobi* genome but only 1.16% of the *C. aquatica* HG genome, and other Ty1/Copia elements including CL4, CL8, CL9, CL11, and CL27 all displayed a similar pattern (Supplemental Data 2). Together, Ty1/Copia retroelements have a higher percentage in *C. lacryma-jobi* than in *C. aquatica* HG. Similarly, some clusters of Ty3/Gypsy also showed genome proportion change, for example, CL2 and CL414 occupies 1.7% and 0.000134% of the *C. lacryma-jobi* genome respectively, while 1.07% and 0.00707% of the *C. aquatica* HG genome respectively. To further verify these results, we conducted FISH on the chromosomes of the two species using CL2 and CL414 as probes (Additional file1: Figure S2). CL2 probe exhibited intense signal in both species. In *C. aquatica* HG, 20 out of 30 chromosomes showed stronger signals than the other 10 chromosomes. CL414 probe only showed faint signal in *C. lacryma-jobi,* while intense signal was observed on *C. aquatica* HG chromosomes*.* Similar to the signal of CL2 probe, 20 out of the 30 chromosomes in *C. aquatica* HG showed stronger signal of CL414 probe than the other 10 chromosomes. The distribution and proportion of Ty1/Copia and Ty3/Gypsy family members were illustrated in Figure 1. Our sequencing results revealed that four families (CL286, CL411, CL429, and CL472) were present only in one species but absent in the other species. We further confirmed the results of CL411 and CL429 by FISH (Additional file1: Figure S3). This varied abundance of the LTR retroelements in the two species may result from sweep or accumulation of the same families, or the rapid change during polyploidization[30].

Our results showed that the satellite DNA made up only 0.60% of the *C. lacryma-jobi* genome in contrast to 4.89% of the *C. aquatica* HG genome. We also found that the most abundant repeats in these two species were completely different, and the proportion of the co-existing satellite repeats varied greatly in these two genomes (Table 3), indicating rapid evolution of satellite repeats in *Coix*. In particular, two distinct satellite repeats (SatS1 and SatS2) made up approximately 2.17% of the *C. aquatica* HG genome (Table 3). The rDNA proportion was similar in the two genomes. The information of other repeat families was presented in the Additional file2.

**Table 3:** **The proportion, length, and location of satellite repeats in** ***C. lacryma-jobi*** **and** ***C. aquatica*** **HG**

Satellite	***C. lacryma-jobi***		***C. aquatica***		Length (bp)	Location
	Rank	GP (%)	Rank	GP (%)		
SatS1	—	—	1	1.34	358	Subtelomere
SatS2	—	—	2	0.83	369	Subtelomere
45S rDNA	1	0.32	6	0.55	–	Chr 1S
SatS3	2	0.29	5	0.57	187	Subtelomere
CentX*	3	0.16	4	0.67	153	Centromere
5S rDNA	4	0.11	9	0.05	–	Chr 4 L
SatS4	5	0.07	7	0.46	184	Subtelomere
SatS5	6	0.06	3	0.79	185	Subtelomere
Telomere	7	0.02	8	0.23	7	Telomere

*CentX is the 153 bp centromere satellite[7]; GP, genome proportion.

### A novel centromeric satellite repeat was identified in *C. aquatica*HG

**Figure 2:**
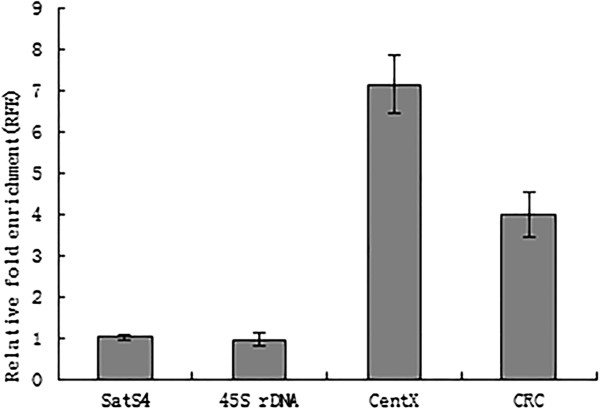
**Relative fold enrichment of centromeric sequences CentX and CRC by the ChIP assay.** Relative fold enrichment of centromeric sequences CentX and CRC by the ChIP assay. SatS4 and 45S rDNA were used as negative controls.

**Figure 3:**
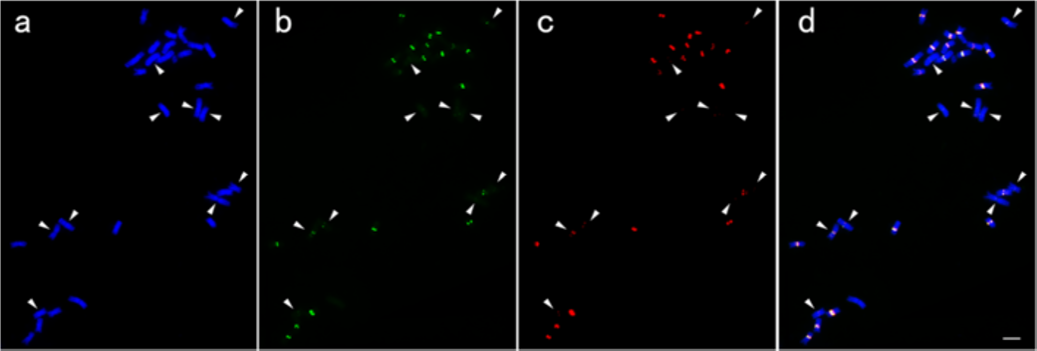
**FISH with the 102 bp and 51 bp fragments of CentX oligonucleotide as probes.** FISH with the 102 bp and 51 bp fragments of CentX oligonucleotide as probes. **(a)** The mitotic metaphase chromosomes of *C. aquatica* HG. The arrowhead pointed the 10 chromosomes less homologues to *C. lacryma-jobi* chromosomes; **(b)** The FISH signals from the 102 bp CentX fragment (green); **(c)** The FISH signals from the 51 bp CentX fragment (red); **(d)** The merged imagine, bar = 5 μm.

It has been demonstrated that ten chromosomes in *C. aquatica* HG with less homology to *C. lacryma-jobi* harbor low copy number of centromeric repeat CentX and a large amount of centromeric retrotransposon CRC[7]. To identify possible new centromeric repeats in these ten chromosomes, we used an OsCenH3 antibody specific for the 30 centromeres in *C. aquatica* HG to perform ChIP-seq assay (Additional file1: Figure S4). Quantitative ChIP PCR showed significant relative enrichment of CentX and CRC in the ChIPed DNA (Figure 2), suggesting that CentX and CRC might be associated with CenH3, the functional centromere marker. In the absence of a reference genome, we mapped ChIP-seq data to the clusters of the *C. aquatica* HG genome for cluster-based repeat identification. The result also showed the significant relative enrichment of CentX and CRC. Besides, we identified a novel CentX variant in *C. aquatica* HG. In contrast to the typical size of 153 bp for CentX, the monomer of this CentX variant had 102 bp and accounted for 1.21% of the total CentX in the ChIPed DNA. According to our whole genome sequencing data, this variant made up 0.45% of the total CentX and 0.003% of the *C. aquatica* HG genome. To test whether this variant is specifically located on the 10 chromosomes less homologous to the *C. lacryma-jobi* chromosomes, we conducted FISH using the sequence of the 102 bp CentX variant and the sequence of the 51 bp missing from the CentX variant but in the typical CentX as separate probes (Figure 3). Both of the 102 bp variant and the 51 bp fragment did not show a different distribution pattern compared with the typical CentX. The extremely faint signals from the two probes showed a similar intensity on the 10 chromosomes less homologous to *C. lacryma-jobi* chromosomes. These results indicate that the 102 bp variant may not be specific to the 10 chromosomes.

### The karyotype of *C. lacryma-jobi*and *C. aquatica*HG

**Figure 4:**
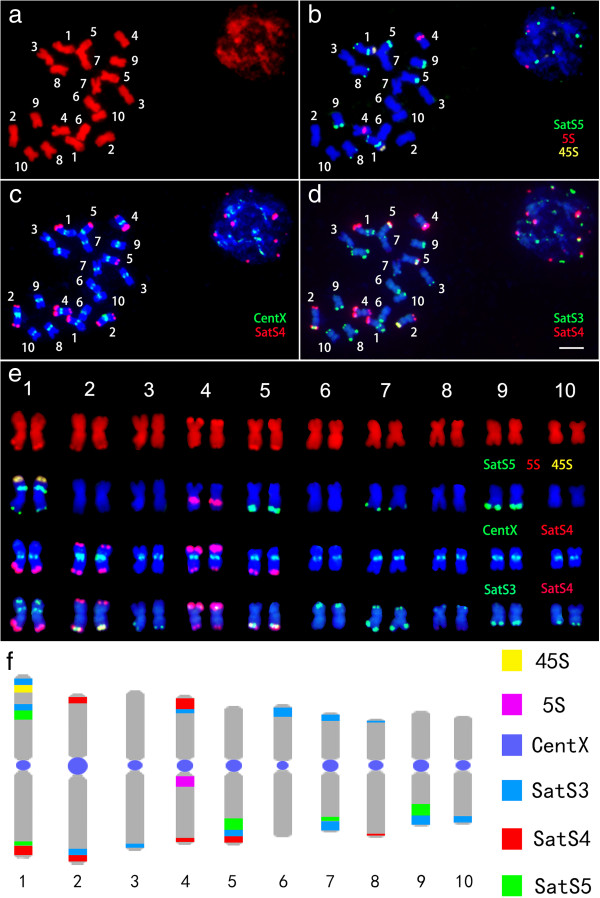
**The karyotype and ideograph for** ***C. lacryma-jobi*** **mitotic metaphase chromosomes.** The karyotype and ideograph for *C. lacryma-jobi* mitotic metaphase chromosomes. **(a)** Mitotic metaphase chromosomes were counterstained with DAPI and pseudocolored in red and chromosomes are numbered according to our karyotyping analysis; **(b)** FISH with the probe cocktail of SatS5 (green), 5S (red) and 45S (yellow); **(c)** The same spread was reprobed with the probe cocktail of CentX (green) and SatS4 (red); **(d)** The same spread was reprobed with the probe cocktail of SatS3 (green) and SatS4 (red); **(e)** Individual chromosomes displayed according to the results in **(a-d)**; **(f)** The Ideograph showing the position and intensity of CentX(blue), SatS3 (light-blue), SatS4 (red), SatS5 (green), 45S rDNA (yellow) and 5S rDNA (purple), bar = 5 μm.

We performed karyotyping analysis on *C. lacryma-jobi* and *C. aquatica* HG using the satellite repeats, 5S rDNA and 45S rDNA as probes (Figure 4). The signal of the centromeric satellite repeat CentX was large on the Chromosomes pair 2, strong on the chromosomes pairs 4, 5, 8 and 9, weak on the chromosomes pairs 1, 3, 7 and 10, and faint on the chromosomes pair 6. The 45S rDNA probe appeared as one large signal at the end of the short arm of the chromosome pair 1. The signal from the 5S rDNA was located at the peri-centromeric region of the long arm of the chromosome pair 4. It is generally agreed that, SatS3, SatS5, and SatS4 signals sequentially approach the telomere at the subtelomeric region. Thus, using these five repeat probes, we expected to identify the individual somatic chromosome in *C. lacryma-jobi*. The signal patterns on *C. lacryma-jobi* chromosomes were shown in Figure 4e. We prepared an ideogram displaying the position and fluorescence intensity of satellite repeats on *C. lacryma-jobi* chromosomes (Figure 4f). The relative length of each chromosome pair was listed in Additional file1: Table S1.

**Figure 5:**
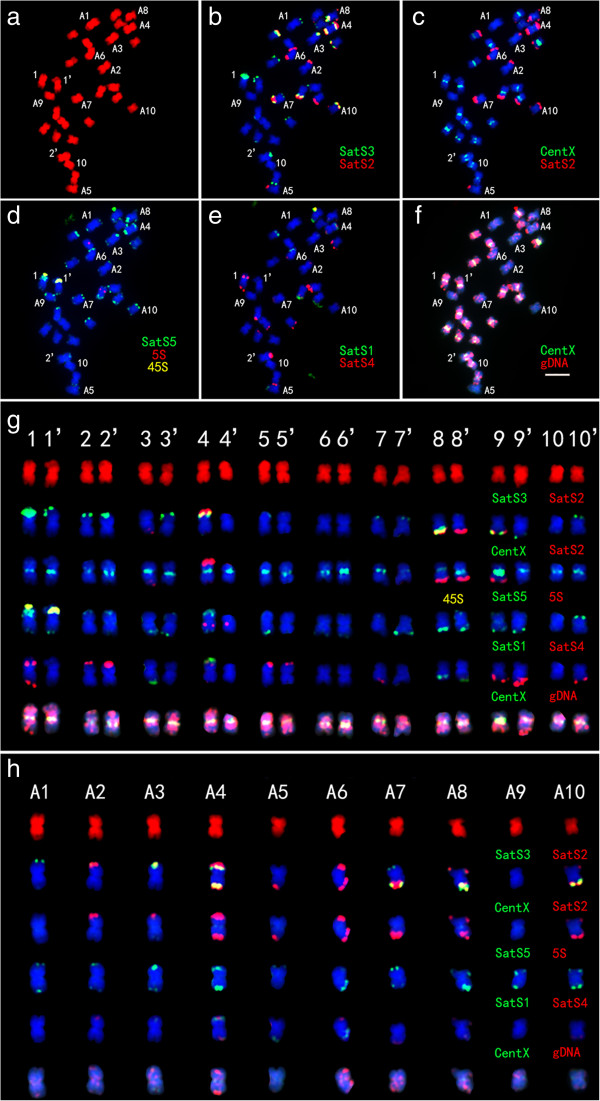
**The karyotype for** ***C. aquatica*** **HG mitotic metaphase chromosomes.** The karyotype for *C. aquatica* HG mitotic metaphase chromosomes. **(a)** The mitotic metaphase chromosomes (numbered from 1 to 10 ) were counterstained with DAPI and pseudocolored in red; **(b)** FISH with the probe cocktail of SatS3 (green) and SatS2 (red); **(c)** A merged imagine with CentX in green and SatS2 in red; **(d)** The same spread was reprobed with the probe cocktail of SatS5 (green), 5S (red) and 45S (yellow); **(e)** The same spread was reprobed with the probe cocktails of SatS1 (green) and SatS2 (red); **(f)** The same spread was reprobed with the probe cocktail of CentX (green) and *C. lacryma-jobi* gDNA (red); **(g)** The twenty chromosomes closely homologous to *C. lacryma-jobi* were separated from figure **(a-g)** and listed in numerical order; **(h)** Ten chromosomes distant to *C. lacryma-jobi* were separated from figure **(a-g)** and listed in numerical order, bar = 5 μm.

**Figure 6:**
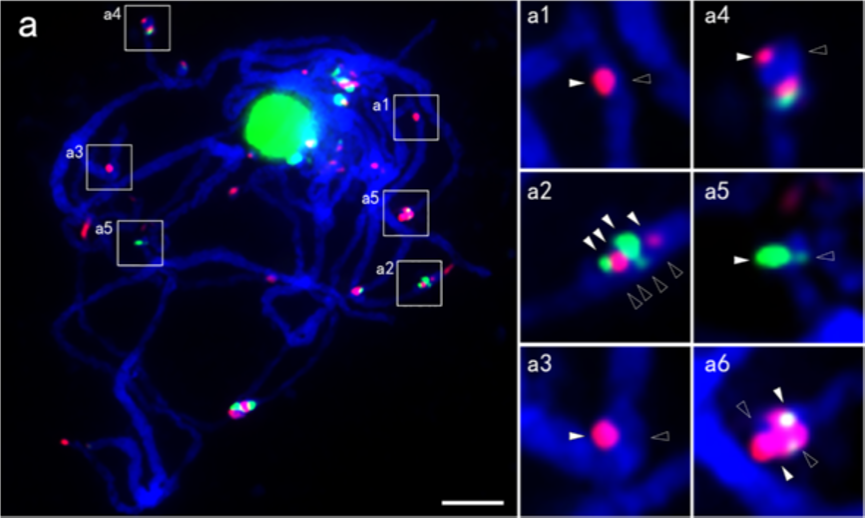
**FISH analysis of asymmetric satellite distribution on pachytene chromosomes of** ***C. aquatica*** **HG.** FISH analysis of asymmetric satellite distribution on pachytene chromosomes of *C. aquatica* HG. The arrowhead pointed FISH signals, and the hollow arrowhead pointed the absence of the signals from the same type of probes or the obvious weaker signals. **(a)** Satellite staining on a pachytene chromosome of *C. aquatica* HG with SatS3 (green) and SatS5 (red) probes. **(a1)** The enlarged image showing an unichromosomal SatS5 signal (red); **(a2)** The enlarged image showing two SatS5 signals (red) and two SatS3 signals (green), with one exceptionally weak SatS3 signal (green, hollow arrowhead) on a homologous chromosome; **(a3)** The enlarged image showing a SatS5 (red) signal on one of the synapsed chromosomes; **(a4)** The enlarged image showing an unichromosomal SatS5 signal (red, arrow head); **(a5)** The enlarged image showing a strong SatS3 signal (green, arrowhead) on one homologous chromosome and a faint one (hollow arrowhead) on the other partner; **(a6)** The enlarged image showing imbalanced SatS5 (red) and SatS3 (green) signals on a set of paired chromosomes, bar = 5 μm.

We used a similar approach to determine the karyotype of *C. aquatica* HG (Figure 5). By using the CentX and *C. lacryma-jobi* genomic DNA probes, we found that 20 out of the 30 chromosomes of *C. aquatica* HG were highly homologous to the chromosomes of *C. lacryma-jobi*. We then further karyotyped these 20 chromosomes by sequential FISH using the following three probe cocktails: 1) 45S rDNA and 5S rDNA; 2) co-existing satellites SatS3, SatS4 and SatS5; 3) SatS1 and SatS2. Signals from the co-existing satellite probe cocktail showed not only different distribution patterns in *C. aquatica* HG compared with *C. lacryma-jobi*, but also a pronounced asymmetric distribution on each chromosome pair (SatS3, SatS4 and SatS5 signals were on Chr. 1, 1’ and 4, 4’). We confirmed this observation by FISH on pachytene chromosomes (Figure 6). Under the condition of enhanced visualization of chromosome pairing, SatS3 and SatS5 signals on the homologous chromosome pairs appeared as a pattern of contrasting presence on one chromosome and absence on the paired one, or large amount on one chromosome and small amount on the paired one (Figure 6).

**Figure 7:**
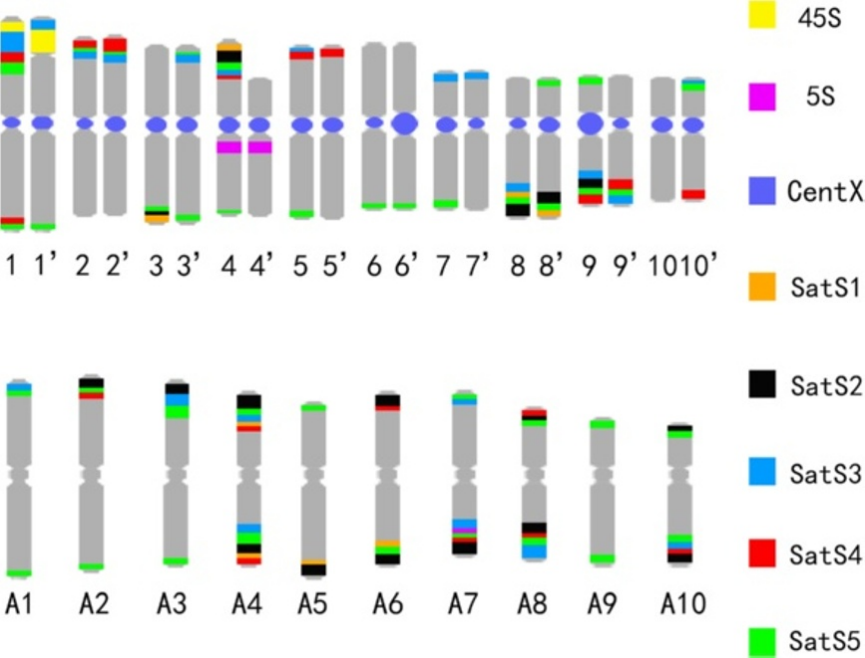
**The ideograph for** ***C. aquatica*** **HG mitotic metaphase chromosomes.** The ideograph for *C. aquatica* HG mitotic metaphase chromosomes. Ideogram showing the position and intensity of SatS1 (tan), SatS2 (black), SatS3 (light-blue), SatS4 (red), SatS5 (green), CentX (blue), 45S rDNA (yellow) and 5S rDNA (purple) on the 20 chromosomes and 10 less homolog chromosomes.

Next, we numbered the remaining 10 chromosomes as A1 to A10 and stained them with the probe cocktails. The signal distribution patterns of the probes on A1-A10 chromosomes were quite different from the patterns on the other 20 chromosomes*.* A faint 5S rDNA signal appeared near the end of the long arm of Chr. A7. SatS5 was absent on chromosomes A5 and A6, and 45S rDNA signal was absent on all the 10 chromosomes, indicating a different origin of this set of chromosomes compared with the other 20 chromosomes. We made a putative ideograph displaying the position and fluorescence intensity of satellites on *C. aquatica* HG chromosomes (Figure 7). The relative length of chromosome pairs was listed in Additional file1: Table S2.

### Determination of the ploidy level of *C. lacryma-jobi*and *C. aquatica*HG

Because certain *Coix* species have ten chromosomes[31], it is possible that *C. lacryma-jobi* is a tetraploid and *C. aquatica* HG is a hexaploid. According to previous reports, *C. aquatica* should be fertile and able to propagate by fertilization[32]. However, our karyotyping analysis revealed 10 chromosome pairs in *C. lacryma-jobi* and 10 paired plus 10 unpaired chromosomes in *C. aquatica* HG, implying the absence of any typical characteristics of a tetraploid or hexaploid genome (Figures 4 and5). We then determined chromosome pairing during metaphase I in *C. lacryma-jobi* and *C. aquatica* HG using CentX and CRC probes. Consistent with the previous report[8], our cytogenetic analysis showed that all the examined *C. lacryma-jobi* meiotic cells had 10 bivalents, while except one cell with an ambiguous trivalent, the rest of the examined *C. aquatica* HG meiotic cells had 10 bivalents and 10 univalents (Table 4). We also analyzed pollen viability of the *C. aquatica* HG using I_2_-KI dying method, and found that the pollen grains were sterile (Additional file1: Table S3). Thus, based on the fact that *C. aquatica* HG is reproduced by vegetative propagation[8] and our findings, we believed that the *C. aquatica* HG might be recently formed hybrid and *C. lacryma-jobi* is a diploidized paleotetraploid species.

**Table 4:** **Statistical summary of bivalents examined in meiotic metaphase I cells of** ***C. lacryma-jobi*** **and** ***C. aquatica*** **HG**

***C. lacryma-jobi***			***C. aquatica***	
10 bivalents	354		10 bivalents with 10 univalents	31
–	–		One ambiguous trivalent	1
Multivalents	0		Multivalents	0
Total	354		Total	32

### Comparative analysis on the repeat sequence in *Coix*, *Zea*and *Sorghum*

**Figure 8:**
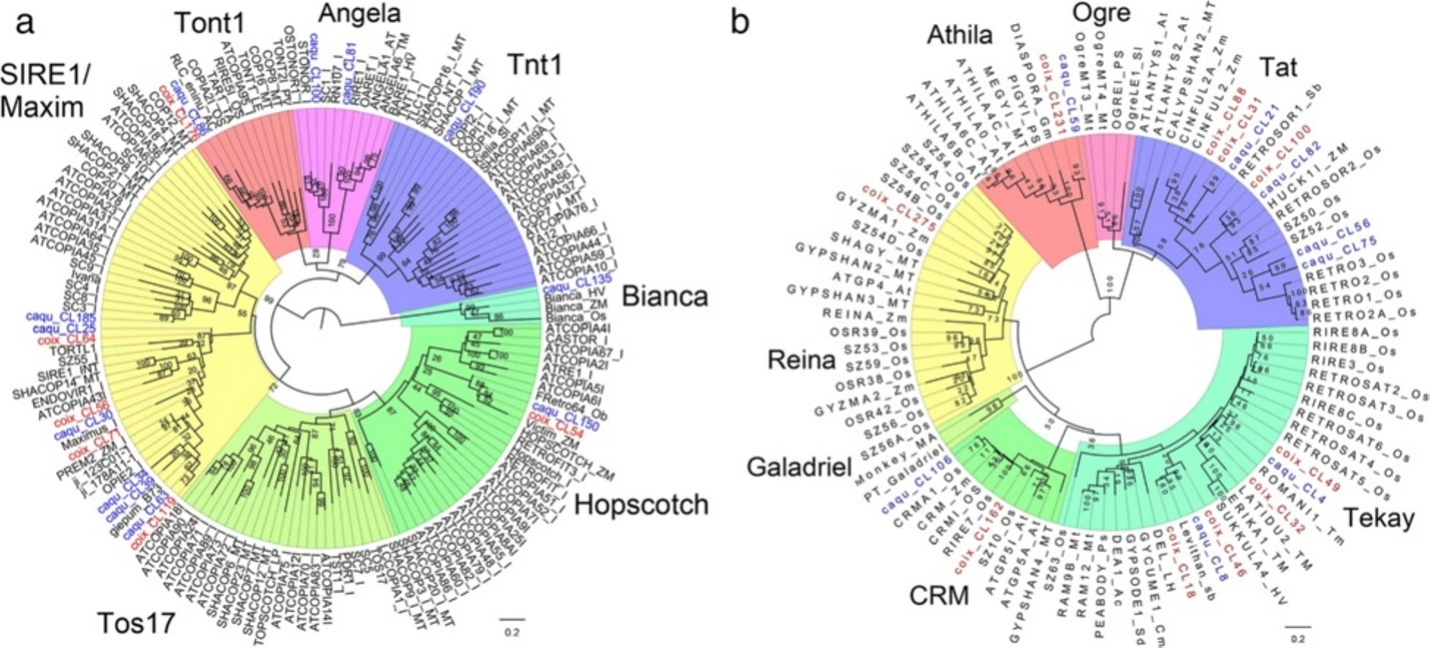
**Phylogenetic relationships of LTR-retrotransposon elements from** ***C. lacryma-jobi*** **and** ***C. aquatica*** **HG.** Phylogenetic relationships of LTR-retrotransposon elements from *C. lacryma-jobi* and *C. aquatica* HG, inferred from Neighbor-joining analysis of the reverse transcriptase encoding domain with bootstrap of 100 replicates. **(a)** The Ty1/Copia families from *C. lacryma-jobi* and *C. aquatica* HG. The elements from *C. lacryma-jobi* and *C. aquatica* HG were in red and blue separately; **(b)** The Ty3/Gypsy families from *C. lacryma-jobi* and *C. aquatica* HG. The elements from *C. lacryma-jobi* and *C. aquatica* HG were in red and blue separately.

We performed classification analysis of LTR for the *C. lacryma-jobi* and *C. aquatica* HG using plant Ty1/Copia clades including Tos17, SIRE1/Maximus, Tnt1, Angela, Tont1, Reina and Bianca[33]. As illustrated in Figure 8a, *Coix* Ty1/Copia elements were distributed in six clades except the Tos17 clade. The most abundant retroelements in *Coix* is the SIRE1/Maximus family members, which is also abundant in maize genome[34]. Our results show that all abundant Ty1/Copia families in *Sorghum* and *Zea* were also present in *Coix,* indicating a close relationship of these three genera.

Additionally, we analyzed seven well characterized Ty3/Gypsy clades including CRM, Hopscotch, Tekay, Galadriel, Athila, Ogre and Tat[33]. We found Tekay, CRM, Reina, Athila, and Tat in *Coix* (Figure 8b). *Huck* in Tekay has been shown to be one of the four most dominant retroelements in *Zea mays*, and a moderate-repetitive DNA in *Sorghum bicolor*[35]. *Leviathan* in Tat was found to be abundant in *Sorghum bicolor*, and moderately abundant in *Zea mays* with no recent activities[35]. We found that *Huck* shared one node with coix_CL100 (0.238%) and caqu_CL82 (0.361%), and *Leviathan* also shared one node with coix_CL18 (0.864%) and caqu_CL8 (1.11%), indicating that the divergence of *Coix* and *Sorghum* might be later than that of *Sorghum* and *Zea*.

**Figure 9:**
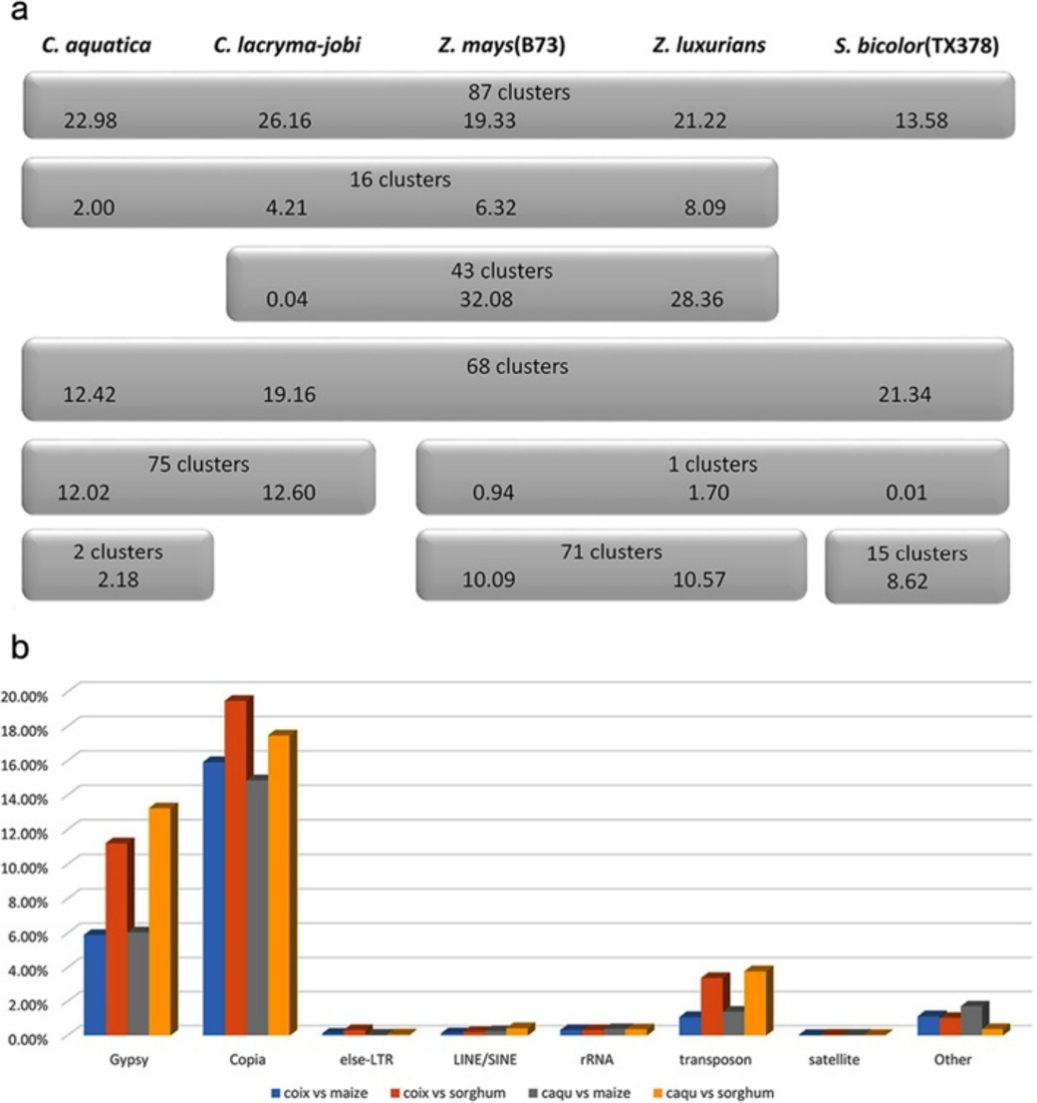
**Comparative analysis of repeat sequences in** ***Zea*** **,** ***Sorghum*** **and** ***Coix.*** Comparative analysis of repeat sequences in *Zea*, *Sorghum,* and *Coix*. **(a)** Distribution of repetitive DNA clusters in five species of *Zea*, *Sorghum,* and *Coix*, focusing on the 9 major distribution patterns and the corresponding numbers of clusters. **(b)** The RepeatMasker annotation by using *Zea mays* and *Sorghum bicolor* as the reference database.

We downloaded paired-end sequences of *Zea mays* (the inbred line B73), *Zea luxurians*, and *Sorghum bicolor* (the inbred line Tx378) fromhttp://sra.dnanexus.com/, and combined all the sequences. We found that repetitive sequences account for 79% of the maize genome and 59.4% of the sorghum genome which is very similar to the previous reports[18, 19]. Distribution of major repeat families in the five species showed that the species-specific clusters occupied 2.18% of the *C. aquatica* HG genome and 8.62% of the sorghum genome, but were not detected in the other three species (Figure 9a). The co-existing clusters made up 22.98% of the *C. aquatica* HG genome, 26.16% of the *Coix lacryma-jobi* genome, 19.33% of the *Zea mays* genome, 21.22% in the *Zea luxurians* genome, and 13.58% of the *Sorghum bicolor* genome. *Coix-Sorghum* co-existing clusters accounted for 12.42% of the *C. aquatica* HG genome, 19.16% of the *Coix lacryma-jobi* genome, and 21.34% of the *Sorghum bicolor* genome. Only a few co-existing clusters were found in *Zea* and *Sorghum. C. lacryma-jobi* and *Zea* shared 43 clusters, however, these clusters only made up 0.04% of the *C. lacryma-jobi* genome. Thus, *C. lacryma-jobi* and *C. aquatica* HG shared more co-existing repeat families with *Sorghum* than with *Zea*. To confirm that *Coix* shared more repeat sequences with *Sorghum* than with *Zea*, we analyzed repeat sequences in *C. lacryma-jobi*, *C. aquatica* HG, maize, and sorghum using the RepeatMasker program (RepBase 20130422) and found that repeat sequences in *Coix* were more similar to those in sorghum than to those in maize (Figure 8b). Therefore, our results corroborate the previous phylogenetic speculation, which shows that *Coix* might be closer to *Sorghum* than to *Zea*[14, 36, 37].

## Discussion

### The dynamic nature of the repetitive sequences in *Coix*

Repetitive sequences play essential roles in genome structuring and evolution, and they usually expand disproportionally to genome enlargement[38]. In the grass family, repetitive DNA accounts for 40% of 466 Mb rice genome[39], 62% of 740 Mb sorghum genome[19], and 82% of 2300 Mb maize genome[18]. In general, the larger a genome size is, the larger is the portion of repeats in the genome. In this study, flow cytometry analysis showed that the genome size of *C. lacryma-jobi* and *C. aquatica* HG, were approximately 1665 Mb and 2335 Mb, respectively (Table 1). By using "cluster-based repeat identification" approach, we reanalyzed the repetitive DNA proportion in *Zea mays* and *Sorghum bicolor* genome as 79% and 59.4%, respectively, which is consistent with the previous reports[18, 19]. We found that *Coix* had a quite high percentage of repetitive DNA: 75.54% for *C. lacryma-jobi* and 72.88% for *C. aquatica* HG *(*Table 2). Notably, our results showed that *C. aquatica* HG with a larger genome than *C. lacryma-jobi* harbored less abundant repetitive DNA compared with *C. lacryma-jobi*. One possibility might be that *C. aquatica* HG may arise from the hybridization of *C. lacryma-jobi* with another relative with less repetitive DNA, indicating a more recent origin of this species. The genome size of *C. aquatica* HG is 1.4-fold of the *C. lacryma-jobi* genome (Table 2). We suspected that the *C. aquatica* HG genome might be still in the process of expanding after original genome fusion. The loss of two tandem repeats (SatS1 and SatS2) and the lower percentage of the co-existing satellites in *C. lacryma-jobi* suggested rapid evolution of this genome (Table 3, Figures 4 and5). The second interpretation might be that the absence of chromosome recombination and rearrangement during meiosis decelerate genome reshuffling in the sterile *C. aquatica* HG. Thirdly, the extent and speed of transposable element expansion may somehow vary in these two species, resulting in the compositional differences of repetitive sequences in the two species[38].

Transposable elements are a major class of repetitive DNA driving genome expansion and speciation[40]. Many families or genera have specific types of transposable elements interspersed in heterochromatic and euchromatic regions[41]. *C. lacryma-jobi* and *C. aquatica* HG consist of typical repetitive DNA in Poaceae family[42]. In this study, the most abundant repetitive elements were Ty1/Copia and Ty3/Gypsy, comprising 68.68% of the *C. lacryma-jobi* genome and 63.68% of the *C. aquatica* HG genome (Table 2). Similar to differential enrichment of Ty1/Copia and Ty3/Gypsy retroelements in *Musa acuminate* genome[43], we found the proportion of Ty1/Copia was moderately higher than that of Ty3/Gypsy in *C. lacryma-jobi* (Table 2). However, Ty3/Gypsy retroelements were more abundant than Ty1/Copia retroelements in *C. aquatica* HG in our study, which is similar to *Zea* and *Sorghum*, *Oryza sativa*, *Spirodela polyrhiza, Brachypodium distachyon,* and *Arabidopsis thaliana*[44]. Inspection of Ty1/Copia and Ty3/Gypsy content of plant genomes in a phylogenetic context reveals no congruence in their content even in closely related species and highlights differences in success of genome occupation[45]. This obvious difference between these two species may be caused by differential amplification or removal of Ty1/Copia and Ty3/Gypsy elements in *C. lacryma-jobi* and *C. aquatica* HG.

Satellite repeats are also a critical class of repetitive DNA in higher eukaryotes[46]. Long stretches of satellite repeats may reside in a special chromosomal locus as a hallmark, functional unit, or a driving force for genome revolution[47]. The centromere specific satellite repeats are particularly important for centromere formation, expansion, and function[48–50]. Consistent with their functional conservation, centromeric satellite repeats are broadly conserved in length, in spite of large variation in their DNA sequences[51]. The size of individual centromeric satellite repeat is 155, 165, 176, and 171 bp in maize, rice, *Brassica*, and human, respectively[52–55]. Very interestingly, we found 153 and 102 bp centromeric satellite repeats in *Coix*. The 153 bp satellite repeat is within the normal range of the length of centromeric satellite repeats, while the 102 bp repeat is the shortest centromeric satellite repeat reported in Poaceae thus far. Similarly, different satellite repeats are also detected in potato[56]. In-depth comparative sequence analysis of five homologous centromeres in two *Solanum* species indicates that centromeric satellite repeats may undergo boom-bust cycles before a favorable repeat is fixed in a population[57]. This may explain why the 153 bp and 102 bp repeats co-exist in the *Coix* centromere. Megabase-sized centromeric satellite repeats in large genomes may trace back to LTR retrotransposon[58, 59], although neocentromeres may function properly without satellite repeats during early stages of centromere evolution[60]. Given that *C. aquatica* HG is sterile and spreads by vegetative propagation, we believe that the evolution of its centromere may be reproductively suppressed. Therefore, the 102 bp variant may represent an evolutionary intermediate of centromeric repeat in *Coix*.

### Ploidy level of *C. lacryma-jobi*and *C. aquatica*HG

*C. lacryma-jobi* (2n = 20) and *C. aquatica* HG (2n = 30) are generally known as tetraploid and hexaploid species, respectively, because the diploid species within the *Coix* genus has been reported to contain 10 chromosomes in its somatic cells[31].The genus *Sorghum* has 25 species with 10, 20, 30, or 40 chromosomes, and these species are classified as diploid plants[61]. Ploidy level of *C. lacryma-jobi* and *C. aquatica* HG remains inconclusive. Karyotyping analysis has been shown to be able to provide direct evidence for homologous chromosome identification in polyploid species by visualizing characteristic chromosomal structures[62]. Taking advantage of a set of powerful satellite repeat markers, we found clear SatS3, SatS4 and SatS5 signal pairs on metaphase chromosomes in the *C. lacryma-jobi* karyotype, which represent the typical feature of diploid karyotypes. Furthermore, we found that 20 chromosomes displayed 10 perfect pairs in pachytene, which was consistent with the 10 bivalents during the meiosis I. Therefore, we speculated that *C. lacryma-jobi* might behave as a diploid species. However, our observation could not rule out the possibility that *C. lacryma-jobi* might be an ancient tetraploid species. During the evolution of many ancient tetraploid species, diploidization often occurs to restructure the genome[63, 64]. Thus, we suspected that the *C. lacryma-jobi* genome, as a tetraploid during its early evolution, might go through subsequent diploidization, similar to what has happened to maize.

In *C. aquatica* HG, GISH with *C. lacryma-jobi* genomic DNA has been shown to identify 20 homologous chromosomes[7]. In this study, the 20 chromosomes also had bright centromeric satellite (CentX) signals (Figure 4). Additionally, some chromosome pairs, such as chromosome 1 and 1’, and chromosomes 4 and 4’, showed asymmetric repeat signals of SatS1, SatS2, SatS3, SatS4 and SatS5 (Figure 4), suggesting that the 20 paired chromosomes may be derived from a recent hybridization of two dissimilar parents and that repeat sequence homogenization may be still an ongoing process[65]. We also found that the other 10 chromosomes have extremely faint signals of centromeric satellite repeats (Figure 5h) compared with the 20 paired chromosomes, and the signal patterns of other satellites on these 10 chromosomes were also different from those on the 20 paired chromosomes in *C. aquatica* HG or in the *C. lacryma-jobi* chromosomes (Figures 4,5,6,7). We found that during meiosis I, 30 chromosomes of *C. aquatica* HG formed ten bivalents and ten univalents (Table 4). In addition, pollen of *C. aquatica* HG was almost completely sterile (Additional file1: Table S3). All these data suggested that the *C. aquatica* HG could be a recently formed hybrid between an octoploid cytotype (2n = 40) related to *C. lacryma-jobi* and a tetraploid cytotype (2n = 20) of a more distantly related *Coix* species (Additional file1: Figure S5). Another possibility is that the hybrid plant might be formed by a diploid (2n = 20) and a different diploid (2n = 20) parent, consequently resulting in a numerically unreduced gamete, hence mimicking a partial autopolyploidization of the second nonhomologous genome.

### The phylogenetic relationship of *Zea*, *Sorghum*and *Coix*was corroborated by comparative repeat sequence analysis

*Zea*, *Sorghum,* and *Coix* are close relatives in Poaceae family, although they independently originated in Central America, Africa, and Southeast Asia, respectively[1]. Previous studies using chloroplast sequences indicate that *Coix* is evolutionarily closer to *Sorghum* than to *Zea*[14, 36], which has been further confirmed by comparative analysis of the *bz* orthologous region[37]. This argument, however, is still questionable due to the absence of evidence from nuclear genome. In our study, we compared all repeat elements of five species from *Zea*, *Sorghum,* and *Coix*. We found that the co-existing repeat families in *Zea* and *Coix* comprised only 16 clusters. However, the *Sorghum* and *Coix* shared 68 clusters. *Zea* and *Sorghum* only had 1 cluster in common (Figure 9a). These lines of evidence from nuclear DNA sequences also support a close evolutionary relationship between *Coix* and *Sorghum* and a relative distant evolutionary relationship between *Zea* and *Sorghum*.

We also identified the most abundant families of LTR-retrotransposon of *Zea* and *Sorghum* in the *Coix* genome, indicating that *Coix*, *Zea* and *Sorghum* may be closely related to one another. It has been shown that *Ji* and *Opie* are the most abundant Ty1/Copia-type retroelements in *Zea mays*, and their copies decrease in *Sorghum bicolor* and *Coix*[35]. *Huck* is an abundant Ty3/Gypsy type retroelement in *Zea mays*[66], but just a moderately repetitive element in *Sorghum bicolor* and *Coix*[35]. Additionally, *Leviathan* is moderately abundant in *Coix* and *Sorghum bicolor* genomes[35]. These results, together with those from repeat sequence comparison, suggest that the dynamics of the genome-wide repeat elements in *Coix*, *Zea* and *Sorghum*, to certain extent, might facilitate the clarification of the evolution relationship of these close species.

## Conclusions

In this study, we used low-coverage sequencing on *Coix lacryma-jobi* L. (2n = 20) and *Coix aquatica* Roxb. HG (2n = 30) to analyze repeat elements. We revealed the genome structure of these two species and large proportional difference of the same repetitive element between two genomes, indicating evolutionary divergence of these two species. The fine karyotyping analysis using satellites as markers and the chromosome pairing of meiotic metaphase I of these two species suggests that *C. lacryma-jobi* is likely a diploidized paleotetraploid species, and *C. aquatica* HG might be an early generation hybrid between *Coix lacryma-jobi* L. and a distantly related species. In addition, the genome-wide repeat sequence comparative analysis of *Zea Mays*, *Zea luxurians*, *Sorghum bicolor,* and *Coix* indicated that *Coix* seem to be closer to *Sorghum* than to *Zea*, which is consistent to previous report. Our results shed new light on the evolution and species differentiation in the Poaceae family*.*

## Methods

### Plant materials

*C. lacryma-jobi* cultivar BJ and *C. aquatica* HG were used in this study. The chromosome number and genomic features of *C. lacryma-jobi* and *C. aquatica* HG were listed in Table 1. BJ is the most important cultivar in north China, it is dry habitat, it’s leaf blades and sheaths are broad and glabrous, it’s false fruits is soft and pyriform. This phenotype is consistent to *C. lacryma-jobi*[15]. The 2n = 30 *C. aquatica* HG is the only *C. aquatica* in China, it was found in northwest of China[16]. It is perennial, aquatic, stoloniferous and rooting from the stolon node, but when grown under field conditions it becomes erect; It’s leaf blades and sheaths are narrow and glabrous, the false fruits is hard and pyriform. This phenotype is consistent to *C. aquatica*[15].

### Genome size estimation

The 1C value of *C. lacryma-jobi* and *C. aquatica* HG *was* measured by flow cytometry with propidium iodide (PI) to stain DNA and *Zea mays* inbred line B73 as the reference plant. Fresh leaves were quickly cut with a sharp knife and treated with the lysis buffer (0.18% Tris, 0.74% Na_2_EDTA, 0.01% spermine, 5.8% KCl, 1.1% β-mercaptoethanol and 0.1% Triton X-100) for 10 minutes. The suspension was filtered with 30 μm nylon mesh, centrifuged at 800 rpm for 5 min. The supernatant was discarded and the pellet was then resuspended in PI (Propidium Iodide, 50 μg · mL^-1^). A flow cytometer (BD, FACSCalibur, USA) was used for the fluorescence measurement, with 10,000 particles measured per run and three runs performed per plant preparation. The genome size was calculated according to the formula: Genome size_Object_ = (mean G1 nuclei fluorescence intensity_Object_/mean G1 nuclei fluorescence intensity_Standard_) Genome size_Standard_. The peak coefficient of variation percentages were all <5.5%.

### DNA extraction and Solexa sequencing

DNA isolation was followed a standard cetyl trimethyl ammonium bromide (CTAB) extraction protocol[67]. DNA was diluted to a final concentration of 200–300 ng/μl for Illumina sequencing. One hundred bp paired-end reads were obtained from HiSeq2000 platform (BerryGenomics).

The sequences for *Zea mays* inbred line B73 SRR088692, *Zea luxurians* accession PI441933 SRR088692, and *Sorghum bicolor* inbred line Tx378 SRR561245 were downloaded from DNAnexus (http://sra.dnanexus.com/).

### Data access

The genome sequencing data files associated with this study have been submitted to NCBI SRA with accession number SRP049558.

### Data analysis

Sequencing data were preprocessed to remove low-quality reads, and then the unpaired reads were discarded. Repeat sequence assembly was performed using a graph-based clustering approach as described by Novak et al.[26] with the cluster size threshold for detailed analysis as 0.005%. Repeat type identification was done by sequence-similarity searches of assembled contigs against Repbase repeatmaskerlibrary using RepeatMasker[68], and by detection of conserved protein domains using RPS-Blast (Reversed Position Specific-Blast)[69]. Satellite monomer within contig sequences were identified using Tandem Repeats Finder[70]. Contigs corresponding to putative mitochondrial and plastid sequences were identified by NCBI blastn and the clusters were eliminated. The genome proportion of each cluster was calculated as the percentage of reads.

To determine the distribution of different repeats between *C. lacryma-jobi* and *C. aquatica* HG, a combined dataset comprising 68,187,510 reads and labeled reads with sample codes were built and the graph-based clustering analysis was performed as previous mentioned. To further analyze the pattern of repeats evolution between subtribe *Maydeae* and *Sorghum*, *Sorghum bicolor* inbred line BTX623, *Zea mays* inbred line B73 and *Zea luxurians* paired-end data were added to build another combined dataset comprising 15,000,000 reads with sample codes. Both datasets were analyzed as described above.

The whole raw data of *C. lacryma-jobi* and *C. aquatica* HG was masked with RepeatMasker[68] using maize and sorghum as reference genomes. The threshold of identity >70% and coverage >50% were used to screen the output. Then the total read number for each annotation was calculated. By comparing the whole and individual repeat family between these two annotations, the relationship of these genera was analyzed.

### ChIP, ChIP-seq, and quantitative ChIP-PCR

Nuclei isolation was performed according to the published protocol[71]. ChIP was performed following standard protocols[72]. Both ChIPed DNA and mock DNA were used for Solexa sequencing (BerryGenomics). ChIP-qPCR was performed to confirm relative enrichment of specific sequences within anti-OsCENH3 precipitated DNA relative to the DNA sample prepared from pre-blood immunoprecipitation 45S rDNA and SatS3, localized at chromosome ends, were used as negative controls to normalize enrichment of each positive amplicon. Quantitative PCR data were analyzed as described previously[73].

### Repeat Identification from the ChIP-seq dataset

To identify repeats associated with CenH3, a set of 2.5 M 100 bp paired-end ChIP-seq reads were mapped to the genomic Graph-based clusters using PatMan program[74], allowing for maximum of three mismatches including two gaps. Based on its best similarity detected among the genomic sequencing reads, each ChIP-seq read was mapped to a maximum of one cluster.

### FISH, GISH, and immunostaining

Mitotic and meiotic chromosome preparation and FISH were performed following published protocols[75] with a minor modification. Root tips of *C. lacryma-jobi* and *C. aquatica* HG were harvested and immediately exposed to nitrous oxide at 2 atm for 2 hours, then fixed in a 3:1 solution of ethanol: glacial acetic acid. DNA probes for each satellite repeat and rDNA were amplified by PCR using *C. lacryma-jobi* and *C. aquatica* HG genomic DNA. Cloning of satellite repeats and rDNA were conducted using primers designed from extracted repeat clusters. The plasmids were labeled with either biotin-16-UTP, digoxigenin-11-dUTP (Roche Diagnostics), or Diethylaminocoumarin-5-dUTP (PerkinElmer) by standard nick translation. An oligonucleotide of (TTTAGGG)_3_ was labeled at the 5’-end with digoxigenin as a FISH probe to detect telomere repeat location. To determine localization of the CentX variant on *C. aquatica* HG chromosomes, FISH was performed with oligonucleotide AATTCAACTGCGAGTTTTTTCGTGCATCGGTGGGAAAAACGGCCCTCCCTCACGAGTTTT from the 102 bp CentX variant labeled at 5’-end with biotin and AAAACTCATGTTTGGTGGGTTTTTGGCACTTTCATTTCCG from the rest of the 51 bp labeled at 5’-end with digoxigenin. Chromosomes were counterstained with 4’, 6-diamidino-2-phenylindole (DAPI) in Vectashield antifade solution (Vector Laboratories).

Images were captured digitally using a Sensys CCD camera (QIMAGING, RETIGA-SRV, FAST 1394) attached to an Olympus BX61 epifluorescence microscope (Tokyo, Japan). Image-Pro plus 6.5 software (Media Cybernetics) was used to measure FISH signals and chromosome parameters. Images were adjusted with Adobe Photoshop 7.0. For karyotype construction, chromosomes in five metaphase cells in each species were measured. Sequential FISH was conducted following published protocol[76]. After first round of FISH and image capture, the slides were washed through a set of PBS (phosphate buffer saline) buffer and ethanol series before hybridized by the second set of probes.

Immunostaining was performed following published protocols[7]. Root tips harvested from plants were fixed in 4% (w/v) paraformaldehyde for 15 min at room temperature. The root tips were squashed on glass slides. The slides were incubated in a humid chamber at 37°C for overnight with primary antibodies against OsCENH3 diluted 1:500 in TNB buffer (0.1 M Tris–HCl, pH 7.5, 0.15 M NaCl, and 0.5% blocking reagent). The slides were incubated with Cy3-conjugated goat anti-rabbit antibody (1:1000) at 37°C for 1 h.

### Pollen viability

To examine pollen viability, fresh pre-flowering panicles from *C. aquatica* HG were collected and stained with 1% iodine-potassium iodide and 1% acetocarmine solutions. More than 400 pollen grains were examined under the Olympus CX41 microscope. Stained pollen grains with a normal size were considered fertile. Small pollen grains with faint staining and empty pollen grains were considered sterile[77].

### LTR retrotransposon family classification

Phylogenetic trees including representative Ty1/Copia and Ty3/Gypsy clades were reconstructed with reference sequences from the previously reported matrices[33]. For each LTR retrotransposon family, one contig covering the RT (reverse transcriptase) domain was used to represent the entire family for phylogenetic analysis. The representative contigs were selected as the most conserved considering their similarity scores with known elements obtained using RPS-BLAST and three alignment profiles: pfam07727, pfam00078 and cd01650. The amino acid sequence within the RT domain was extracted from alignment results of RT boundaries using a custom Python script. Phylogenetic trees were constructed using the Neighbor-joining method and bootstrapped with 100 replicates using the MEGA5.

## Electronic supplementary material

Additional file 1:**Includes Table S1-S3 and the figure legends for Figure S1-S5.**(DOCX 1 MB)

Additional file 2:**List of the annotation and genome proportion of the cluster of the comparative analysis.**(XLSX 26 KB)

## Abbreviations

FISH: Fluorescent in situ hybridization
GISH: Genomic in situ hybridization
Gb: Giga base pairs
Mb: Mega base pairs
bp: Base pair
RT: Reverse transcriptase
ChIP: Chromatin immunoprecipitation.
