# Supplementary material for: Differential genome evolution and speciation of Coix lacryma-jobi L. and Coix aquatica Roxb. hybrid guangxi revealed by repetitive sequence analysis and fine karyotyping
Source: BMC Genomics. 2014 Nov 25;15(1):1025. doi: 10.1186/1471-2164-15-1025 (PMC4256728; doi:10.1186/1471-2164-15-1025)
Supplement: Supplementary file 1 — Additional file 1:Includes Table S1-S3 and the figure legends for Figure S1-S5.(DOCX 1 MB) [file 12864_2014_6718_MOESM1_ESM.docx]

**Figure S1 - The flow histogram of *Zea mays, C. lacryma-jobi* and *C. aquatica* HG**

**
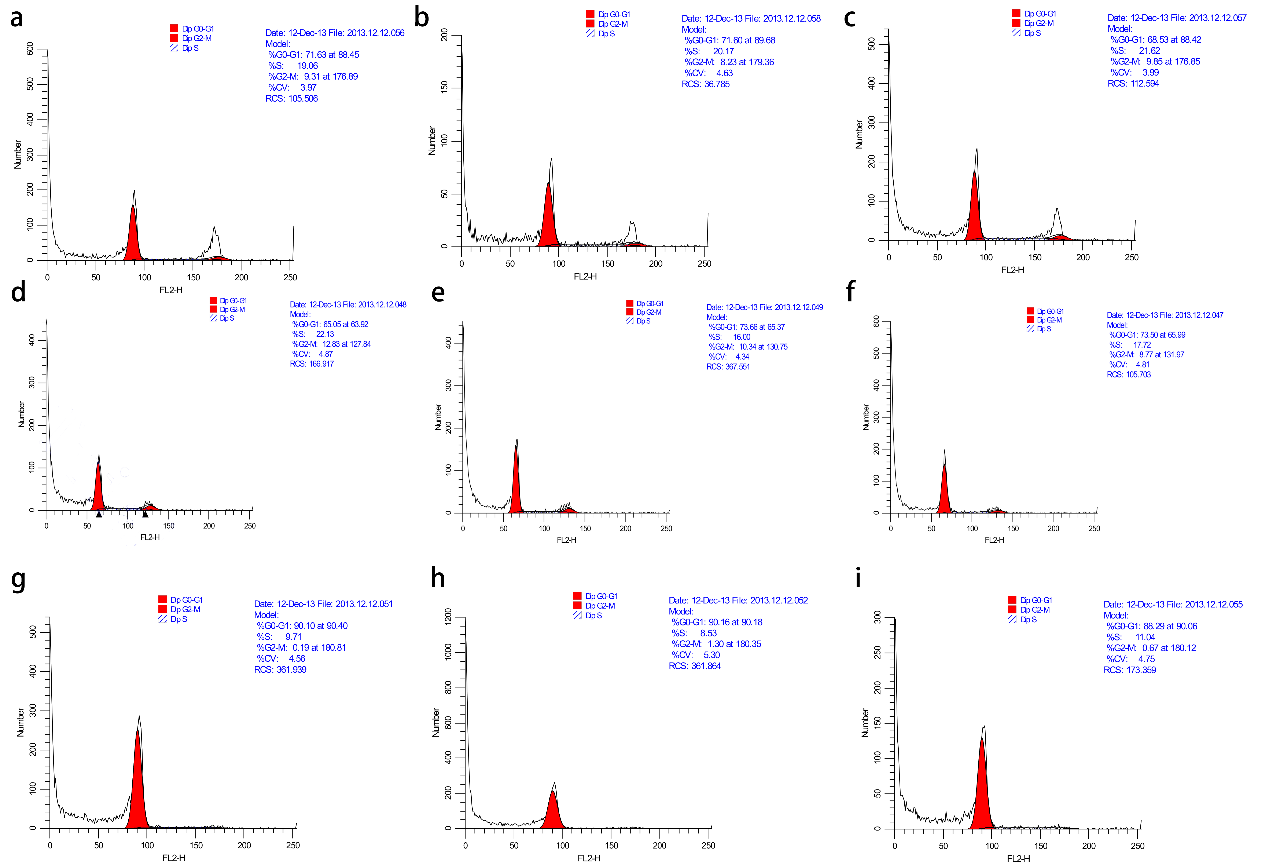
**

Figure S1. The flow histogram of *Zea mays, C. lacryma-jobi* and *C. aquatica* HG. (a-c) Three biological repetitions of *Zea mays*; (d-f) Three biological repetitions of *C. lacryma-jobi*; (g-i) Three biological repetitions of *C. aquatica* HG*.*

**Figure S2 - The distribution of two LTR retrotransposon showing great change of the genome proportion between these two genomes on the mitotic chromosomes of *C. lacryma-jobi* and *C. aquatica* HG**


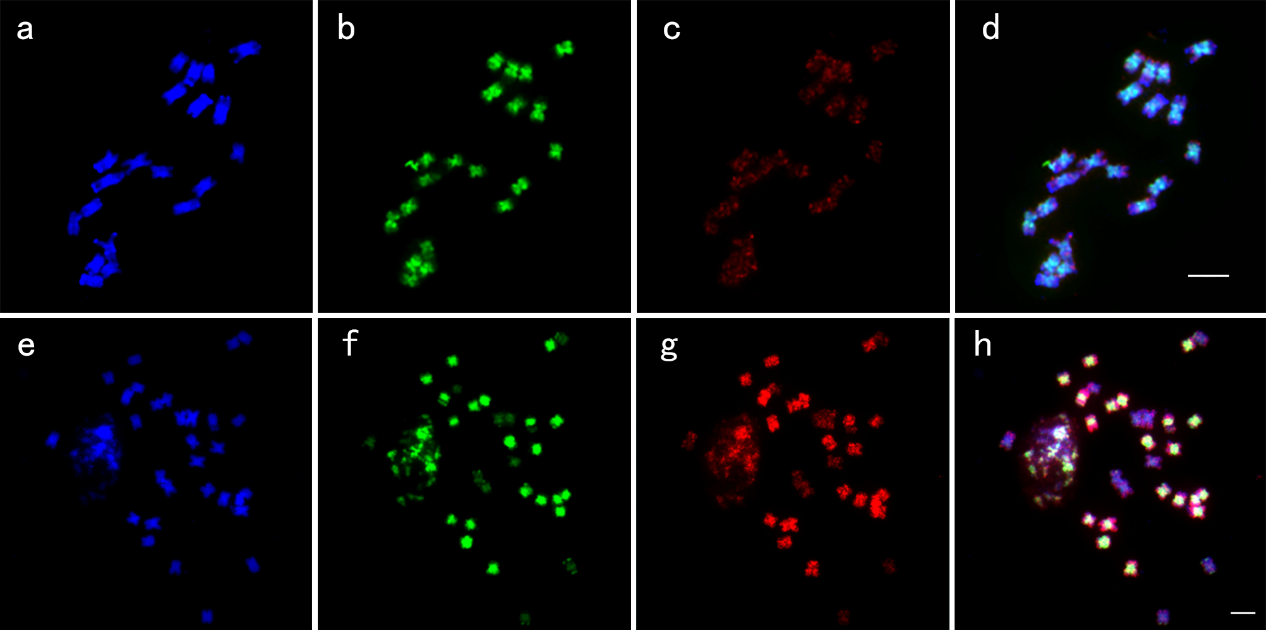


Figure S2. The distribution of the distinct LTR retrotransposon on the mitotic chromosomes of *C. lacryma-jobi* and *C. aquatica* HG. (a) The mitotic chromosomes of *C. lacryma-jobi* were counterstained with DAPI and pseudo-colored in blue; (b) The signal of CL2 (green) on the chromosomes of *C. lacryma-jobi*; (c) The signal of CL414 (red) on the chromosomes of *C. lacryma-jobi*; (d) Merged signals; (e) The mitotic chromosomes of *C. aquatica* HG; (f) The signal of CL2 (green) on the chromosomes of *C. aquatica* HG; (g) The signal of CL414 (red) on the chromosomes of *C. aquatica* HG; (h) Merged signals; bar = 5µm.

**Figure S3 - The distribution of two distinct LTR retrotransposon on the mitotic chromosomes of *C. lacryma-jobi* and *C. aquatica* HG**


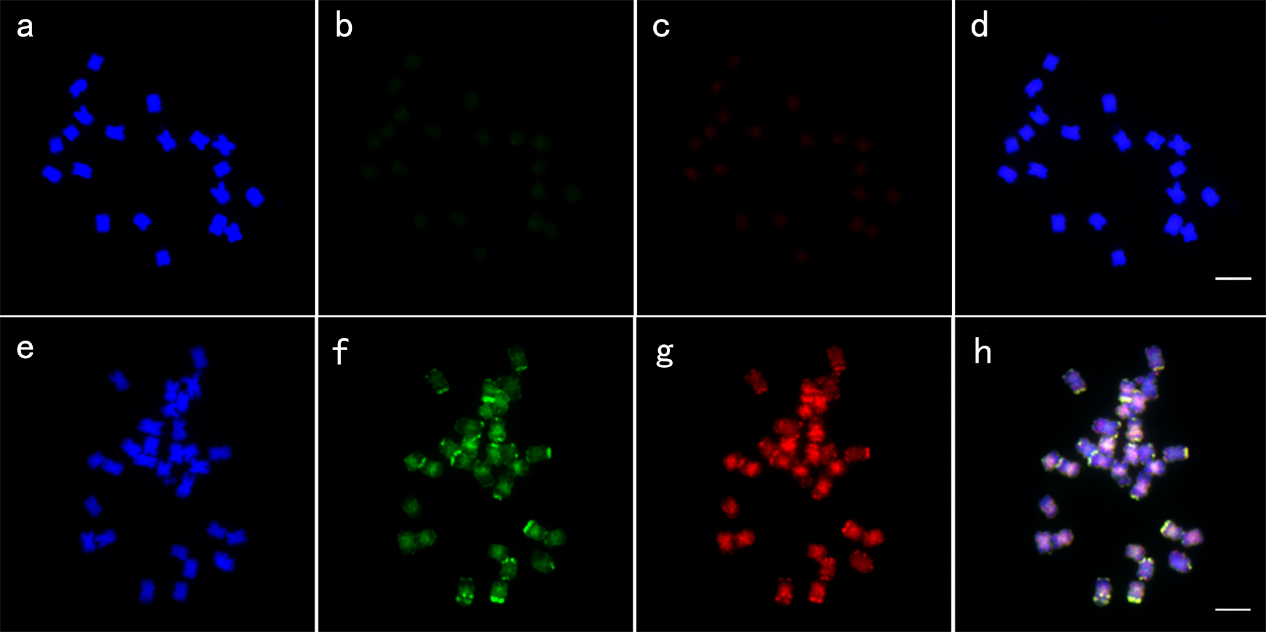


Figure S3. The distribution of the distinct LTR retrotransposon on the mitotic chromosomes of *C. lacryma-jobi* and *C. aquatica* HG. (a) The mitotic chromosomes of *C. lacryma-jobi* were counterstained with DAPI and pseudo-colored in blue; (b) The signal of CL429 (green) on the chromosomes of *C. lacryma-jobi*; (c) The signal of CL411 (red) on the chromosomes of *C. lacryma-jobi*; (d) Merged signals; (e) The mitotic chromosomes of *C. aquatica* HG; (f) The signal of CL429 (green) on the chromosomes of *C. aquatica* HG; (g) The signal of CL411 (red) on the chromosomes of *C. aquatica* HG; (h) Merged signals; bar = 5µm.

**Figure S4 - Immunofluorescence on mitotic chromosomes of *C. aquatica* HG using OsCENH3 antibody**


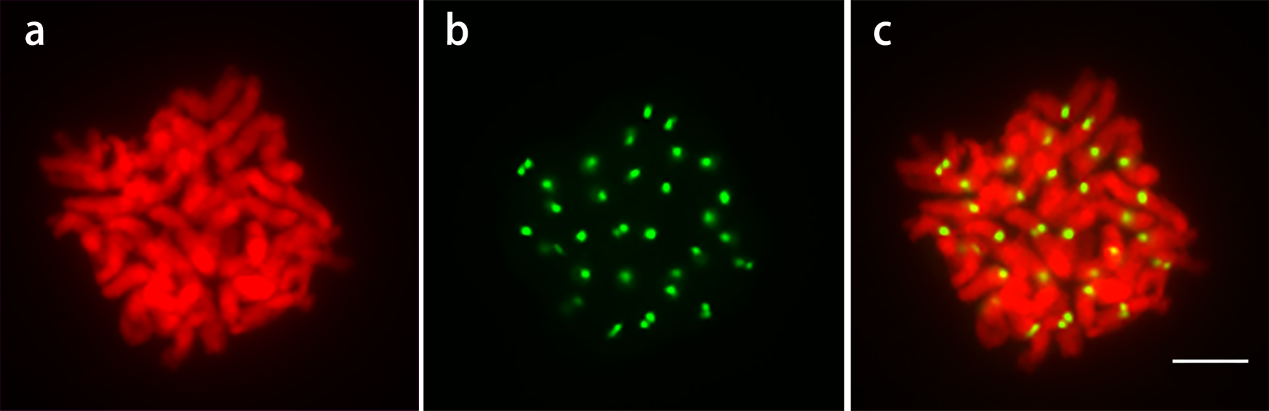


Figure S4. Immunofluorescence on mitotic chromosomes of *C. aquatica* HG using OsCENH3 antibody. (a) The chromosomes were counterstained with DAPI and pseudo-colored in red; (b) Immunofluorescence signals of the anti-OsCENH3 antibodies; (C) Merged signals, bar = 5µm.

**Table S1 - Relative length and arm ratio of mitotic metaphase chromosomes of *C. lacryma-jobi***

| Chr. | Relative length^a^ | Arm ratio^b^ |
| --- | --- | --- |
| 1 | 13.16±0.28 | 1.03 |
| 2 | 12.27±0.40 | 1.35 |
| 3 | 11.43±0.27 | 1.14 |
| 4 | 10.68±0.21 | 1.12 |
| 5 | 9.94±0.36 | 1.34 |
| 6 | 9.47±0.37 | 1.17 |
| 7 | 8.49±0.43 | 1.55 |
| 8 | 8.56±0.35 | 1.25 |
| 9 | 8.25±0.27 | 1.11 |
| 10 | 7.76±0.30 | 1.20 |

^a^ Relative length: 100 × (chromosome length/total complement length).

^b^ Arm ratio: length of the long arm/length of the short arm.

**Table S2 - Relative length and arm ratio of mitotic metaphase chromosomes of *C. aquatica* HG**

| Chr. | Relative length^a^ | Arm ratio^b^ |
| --- | --- | --- |
| 1 | 12.40±0.51 | 1.13 |
| 2 | 11.30±0.91 | 1.11 |
| 3 | 10.20±1.00 | 1.25 |
| 4 | 10.02±1.25 | 1.28 |
| 5 | 10.07±0.53 | 1.20 |
| 6 | 9.88±0.33 | 1.28 |
| 7 | 10.27±1.03 | 1.30 |
| 8 | 8.70±0.63 | 1.65 |
| 9 | 8.67±0.93 | 1.35 |
| 10 | 8.49±1.12 | 1.35 |
| 1’ | 12.97±0.63 | 1.05 |
| 2’ | 11.44±0.49 | 1.09 |
| 3’ | 11.25±0.68 | 1.34 |
| 4’ | 9.02±0.90 | 1.45 |
| 5’ | 10.30±0.38 | 1.28 |
| 6’ | 9.65±0.37 | 1.10 |
| 7’ | 8.89±0.60 | 1.63 |
| 8’ | 8.78±0.86 | 1.82 |
| 9’ | 9.09±0.68 | 1.49 |
| 10’ | 8.61±0.83 | 1.17 |
| A1 | 12.57±0.55 | 1.20 |
| A2 | 11.33±1.63 | 1.16 |
| A3 | 10.97±0.27 | 1.10 |
| A4 | 10.76±0.25 | 1.29 |
| A5 | 10.15±0.76 | 1.05 |
| A6 | 9.91±0.34 | 1.21 |
| A7 | 9.48±0.98 | 1.18 |
| A8 | 8.98±0.82 | 1.17 |
| A9 | 7.77±0.49 | 1.38 |
| A10 | 8.09±1.15 | 1.87 |

^a^ Relative length: 100 × (chromosome length/total complement length).

^b^ Arm ratio: length of the long arm/length of the short arm.

**Table S3 - The pollen viability of *C. aquatica* HG*.***

| Type | Number |
| --- | --- |
| Faintly stained | 434 |
| Irregular shape | 62 |
| Fertile | 0 |
| Total | 496 |

**Figure S5 - Schematic illustration for the formation of *C. aquatica* HG.**


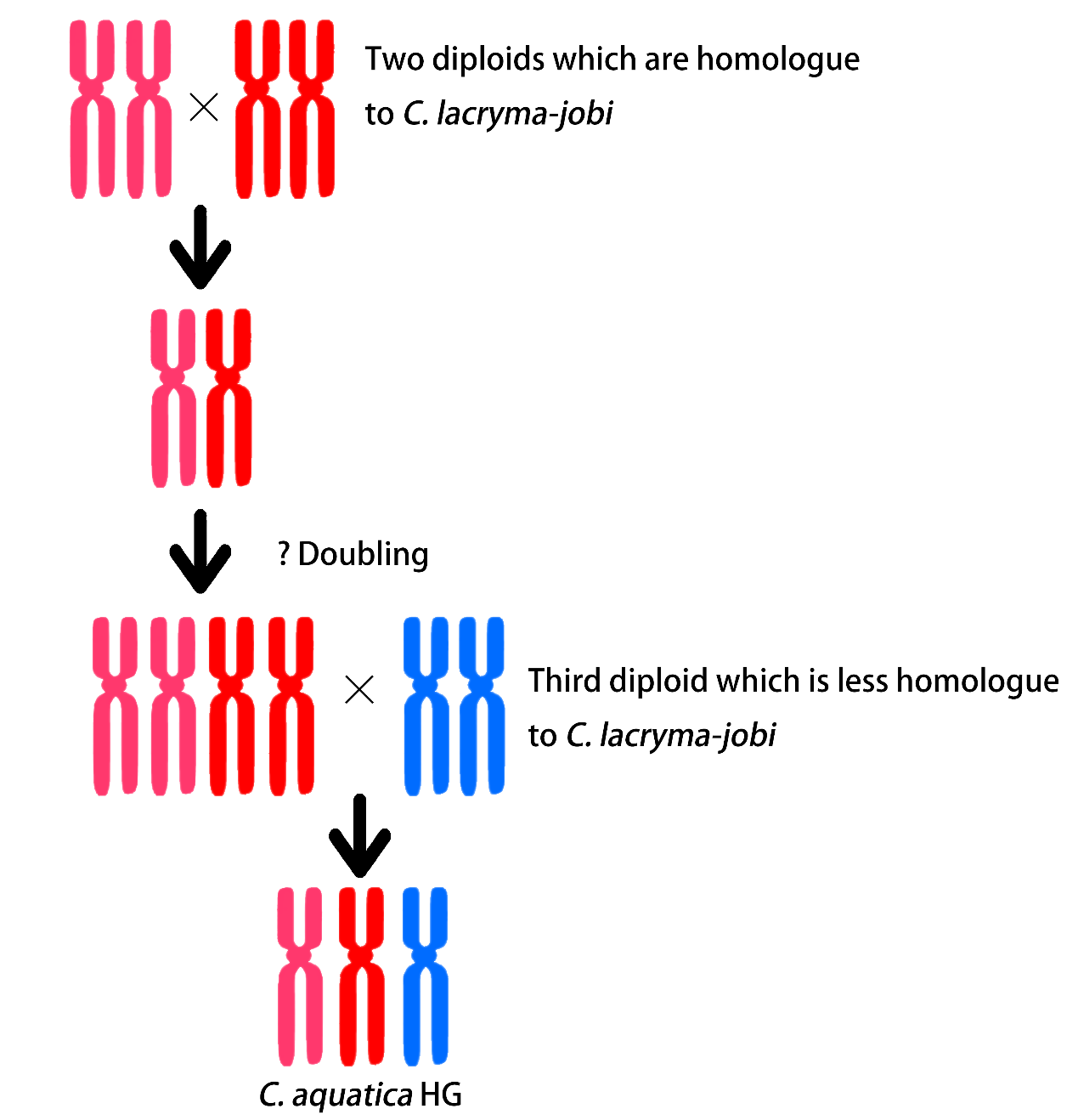


Figure S5. Schematic illustration for the formation of *C. aquatica* HG. The light red and the red chromosomes stand for two genomes which are highly homologue to *C. lacryma-jobi*, the blue chromosomes stands for the genome which is less homologue to *C. lacryma-jobi*.
